# BenchRep-T: A Systematic Evaluation of T-Cell Repertoire-Based Disease Diagnostics

**DOI:** 10.64898/2026.06.09.727013

**Published:** 2026-06-10

**Authors:** Chiho Im, Liel Cohen-Lavi, Alejandro Buendia, Anshul Kundaje, Scott D. Boyd

**Affiliations:** 1 Department of Computer Science, Stanford University; 2 Department of Pathology, Stanford University; 3 Department of Biomedical Data Science, Stanford University; 4 Department of Genetics, Stanford University

## Abstract

Adaptive immune receptor repertoire sequencing data has emerged as a promising potential modality for disease diagnosis, relying on computational methods to analyze T-cell receptor (TCR) sequences from an individual’s blood sample. Published methods rely on different cohorts, data preprocessing pipelines, and evaluation metrics, making direct comparison across methods challenging. We present BenchRep-T, a unified benchmark that standardizes multiple publicly available TCR repertoire datasets and evaluates nine computational approaches, spanning statistical enrichment of shared sequences, feature-engineered ensembles, deep learning, and sequence clustering. BenchRep-T evaluates methods on four tasks: disease classification across conditions, performance scaling under restricted sequence-sampling depth, recovery of known antigen-specific driver sequences, and evaluation of sensitivity to demographic confounding. Under controlled evaluation, simple baselines prove competitive, with tree-based models trained on V- and J-gene usage and short sequence motifs approaching the classification performance of more complex methods. Our findings underscore the complexity of modeling TCR repertoire data, and show that no single method dominates across all tasks. BenchRep-T provides a framework for rigorous and reproducible evaluation of TCR repertoire classification methods to accelerate the development of immune repertoire-based diagnostics.

## 1 Introduction

B and T lymphocytes are the principal cells of the adaptive immune system, with each cell expressing a unique surface receptor—the B-cell receptor (BCR) or T-cell receptor (TCR)—generated by recombination of gene segments. Upon binding an antigen, such as a fragment from a pathogen protein, the cell proliferates to generate a clone of daughter cells sharing the same receptor genetic rearrangement. The adaptive immune receptor repertoire (AIRR) thus encodes a record of past and ongoing immune exposures, providing biomarkers for diverse clinical states [1, 2, 3].

Immune receptor repertoires are highly diverse both within and between individuals; the potential sequence space exceeds 10^18^, with each individual sampling approximately 10^8^–10^10^ distinct receptors of a given type [1, 2]. Overlap of sequences between individuals is low, even when they have had similar immunological exposures, and demographic factors as well as genetic background can influence receptor repertoires. AIRR data has been challenging to generate at scale, requiring experimental and computational infrastructure; most studies have been modest in cohort size relative to the underlying complexity of the repertoire. High-throughput sequencing of the TCR *β* chain (TCR*β*) has nonetheless identified disease-associated signatures across chronic viral infection [4], autoimmunity [5, 6], tuberculosis progression [7], and acute viral infection or vaccination [8, 9], suggesting that TCR*β* repertoires can support disease-specific prediction tasks. A central challenge is that only a small and typically unknown subset of receptors in a sample is relevant to any given immune condition.

Classification of TCR repertoires often makes use of gene segment composition and the complementarity-determining region 3 (CDR3) sequences that are formed by the gene segment junctions, and encode the most diverse part of the TCR. The CDR3 is the primary determinant of specific target antigen recognition [1, 2]. Three broad TCR repertoire classification paradigms have been widely used: statistical identification of enriched sequence patterns or “public” clones shared between individuals [4, 10, 11, 12, 13], ensemble methods combining analysis of repertoire-level features and detection of shared sequences [14, 9], and deep learning methods that learn sequence representations directly from data [15, 16]. AIRR datasets are affected by the experimental protocols chosen for library generation, as well as biological variation [17]. Controlled comparisons of classification methods have been limited, and have varied in many regards: cohorts, train/test splits, preprocessing of the underlying AIRR files, baselines, and experimental and sequencing pipelines [13, 15, 18]. While prior studies have considered some of these questions in specific settings, they have not been examined systematically across diverse method families under a common evaluation protocol.

**Figure 1:**
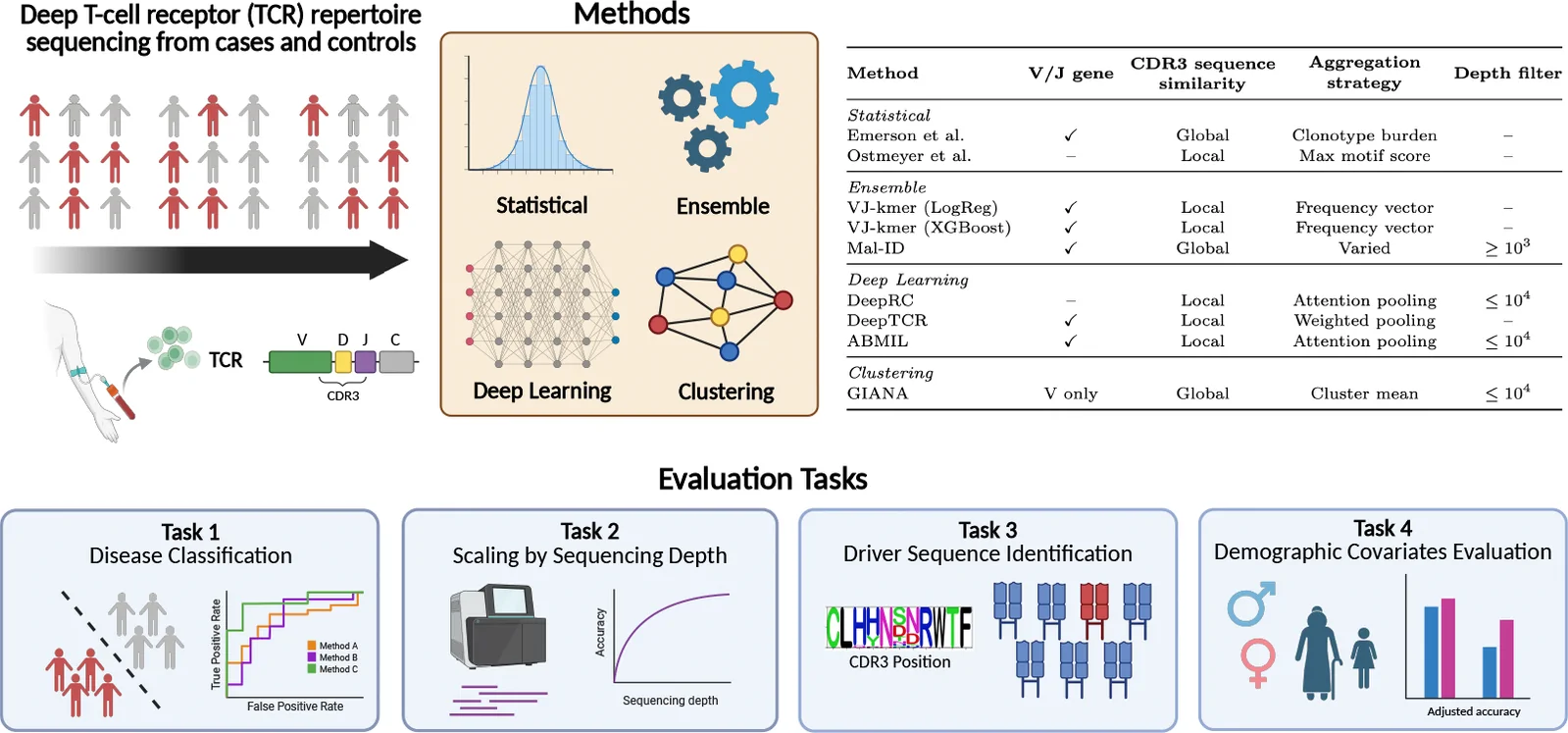
BenchRep-T evaluation framework. Deep TCR repertoire datasets are aggregated from seven disease states and healthy samples and used to evaluate computational approaches on four tasks (**bottom**). Table of method characteristics (**top right**): usage of V/J genes, CDR3 sequence-level feature comparison strategy, repertoire-level aggregation strategy, and repertoire-depth filtering imposed by each method during training (if any). Global denotes whole-sequence comparison (exact clonotype identity, Hamming distance, protein LM embeddings, or sequence alignment); local denotes usage of sub-sequence features (biochemical motifs, gapped *k*-mers, or convolutional windows in deep learning methods). Schematic created with BioRender.com.

We address this need with **BenchRep-T**, a unified evaluation framework for TCR*β*-based disease diagnostics (Figure 1). BenchRep-T harmonizes public data from cohorts described in Zaslavsky et al. [9], containing 550 TCR*β* specimens covering five disease and vaccine conditions plus healthy controls, and three additional cohorts of 431 individuals (T1D [6], tuberculosis progression [7], and rheumatoid arthritis [5]). The combined data are evaluated with nine published or custom-designed TCR repertoire classification methods under identical inputs and identical preassigned cross-validation folds. We define four evaluation tasks: (i) *disease classification* across seven diseases, in single-cohort and cross-cohort settings, (ii) *sequencing-depth scaling*, evaluating performance after repertoire downsampling to fixed depth, (iii) *driver sequence identification*, testing per-sequence rankings to recover known antigen-specific TCRs from public datasets [19, 8], and (iv) *demographic-confounding analysis*, testing the impact on classification performance of demographic structure rather than repertoire-derived disease signal. We also address computational efficiency and resource requirements by benchmarking method runtimes, CPU memory consumption, and GPU memory consumption.

To our knowledge, BenchRep-T is the first benchmark to compare published TCR repertoire classification methods with a standardized multi-cohort dataset, evaluating performance along complementary dimensions. BenchRep-T thus makes published methods easier to compare and provides a reusable and extensible dataset and framework for benchmarking future TCR repertoire classification methods.

## 2 Datasets and Preprocessing

BenchRep-T uses TCR*β* repertoire data from multiple sources [9, 20]. All repertoires were derived from peripheral blood samples and harmonized with common preprocessing so that every benchmarked method received identical inputs. Details are provided in Section B.1.

### 2.1 TCR Repertoire Data Sources

#### Mal-ID.

Mal-ID [9] aggregates bulk BCR (IgH) and TCR*β* immunosequencing data from multiple clinical studies and medical centers, generated with a standardized sequencing protocol. For BenchRep-T TCR-based classification, we use its 550 TCR*β* specimens (542 participants), after excluding 66 BCR-only specimens. The TCR data comprise 91,711,135 sequences from 197 Healthy/Background controls, and five disease or vaccine conditions: 98 **HIV**, 96 **Type 1 Diabetes**, 64 **Lupus**, 58 **COVID-19**, and 37 **Influenza** vaccine specimens. A subset of specimens includes participant-level demographic metadata (age, sex, and self-reported ancestry), shown in Figure A.1.

#### immunoSEQ cohorts.

We additionally include three publicly available TCR*β* datasets obtained using the Adaptive immunoSEQ platform [20], containing a total of 60,258,574 sequences: (i) a **Type 1 Diabetes (T1D)** cohort [6] of 197 specimens (172 T1D, 25 healthy controls), (ii) a **Tuberculosis (TB) progression** cohort [7] of 140 specimens from latently infected individuals, split into 63 progressors and 77 non-progressing controllers, and (iii) a **Rheumatoid Arthritis (RA)** cohort [5] of 94 specimens (74 RA, 20 healthy controls).

### 2.2 Disease Coverage and Evaluation Protocol

T1D is represented in both the Mal-ID and Mitchell et al. [6] datasets. We combined these into a single dataset, exposing each classification method to data from two distinct collection protocols and experimental batches within the same task. Each dataset is partitioned into three disjoint cross-validation folds, with the same folds reused for all BenchRep-T tasks. Mal-ID specimens use the predefined 3-fold splits released with Mal-ID [9]. Each immunoSEQ cohort is independently partitioned into three folds stratified by disease label. To support reproducibility and future method development and benchmarking, we release the scripts producing the cleaned datasets, together with the benchmark splits used throughout BenchRep-T.

Disease classification is formulated as a binary prediction problem within each dataset, using the dataset-specific comparator group: healthy/background controls for the four Mal-ID diseases, pooled healthy controls for the T1D and RA cohorts, and TB progressors versus non-progressing controllers in the TB cohort. For all disease-classification evaluations, we report area under the receiver operating characteristic curve (AUROC) and area under the precision–recall curve (AUPRC). AUROC and AUPRC are computed on out-of-fold predictions pooled across the three CV folds.

## 3 Benchmarked Methods

We evaluate nine methods that together span the major paradigms of repertoire-based disease classification, ranging from interpretable statistical tests to end-to-end deep learning. Each is reimplemented in a unified framework so that all methods consume the same AIRR-formatted inputs and are evaluated as defined in Section 2. Full details are described in Section B.2.

### 3.1 Statistical Disease-signature Methods

**Emerson et al. (2017)** [4] identifies (V gene, J gene, CDR3) triplets that are differentially present between cases and controls via a Fisher’s exact test, forming a diagnostic set of disease-associated clones. Class-conditional Beta-Binomial distributions are fit to each subject’s diagnostic burden (count of clones from this set) conditioned on repertoire size, and test specimens are scored by their log-posterior odds. **Ostmeyer et al. (2019)** [10] represents each repertoire as a bag of CDR3 4-mers, encoding each 4-mer by its Atchley-factor representation augmented with its clone-size-weighted abundance. A logistic regression is trained under a max-aggregation MIL objective, so that each bag’s gradient flows only through its top-scoring “witness” 4-mer.

### 3.2 Ensemble of Engineered Repertoire-level Representations

**V/J gene + gapped k-mer (VJ-kmer) ensemble** represents each repertoire with two relative-frequency feature dictionaries: V- and J-gene usage, and CDR3 4-mers plus their single-position gapped variants. Each is fed to an *L*_1_-penalized logistic regression, and the two sub-model probabilities are combined as a tuned weighted average. We additionally evaluate an XGBoost variant that replaces each base learner with a gradient-boosted tree classifier while preserving the same feature dictionaries. **Mal-ID (2025)** [9] is an ensemble framework that combines three complementary repertoire representations: (i) a composition model based on normalized V/J gene usage, (ii) a convergent-clustering model that groups sequences by V/J gene and CDR3 length and trains a classifier on disease-enriched cluster counts, and (iii) a protein language model pipeline that embeds CDR3s with ESM-2 [21] and aggregates V-gene-specialized sequence-level scores to the specimen level.

### 3.3 Clustering-based Methods

**GIANA (2021)** [11] encodes each CDR3 into a fixed-dimensional vector using a BLOSUM-derived substitution embedding and clusters training sequences by isometric distance. Each cluster is assigned a disease score based on the fraction of training sequences originating from the target disease. A test repertoire is scored by averaging the disease scores of its assigned TCR clusters, with unassigned sequences omitted.

### 3.4 Deep Learning-based Methods

As a learned counterpart to the gapped-*k*-mer ensemble, we implement an **Attention-Based MIL Model (ABMIL)** [22] that replaces hand-designed features with end-to-end learned embeddings. Each CDR3 sequence is encoded with a convolutional neural network and combined with learned V/J-gene embeddings to produce per-sequence features, which are aggregated into a repertoire-level representation using gated attention. **DeepRC (2020)** [15] is a multiple-instance repertoire classifier based on modern Hopfield networks. CDR3 sequences are encoded with convolutional features and aggregated using a Hopfield-style attention mechanism that prioritizes disease-associated sequences within each repertoire. Amino acid and positional features are passed through a three-layer 1-D CNN and the top attention-weighted sequence embeddings are pooled for repertoire classification. **DeepTCR (2021)** [16] is a convolutional multiple-instance learning framework. Sequence embeddings derived from CDR3s and V/J-gene identities are mapped into a learned concept space, and repertoire-level representations are formed through frequency-weighted aggregation of sequence concept activations.

## 4 Evaluation Approaches and Results

BenchRep-T evaluates methods on four tasks: (i) **Disease Classification Benchmarking**, classification performance for each method for all 7 diseases, (ii) **Sequencing Depth Scaling**, how predictive performance changes as fewer sequences per specimen are available, (iii) **Driver Sequence Identification**, prioritization of known antigen-specific TCRs using a method’s per-sequence scores, and (iv) **Demographic Confounding Analysis**, the extent to which a method’s predictive signal may reflect participant demographics rather than disease-associated repertoire biology.

### 4.1 Disease Classification Benchmark

**Figure 2:**
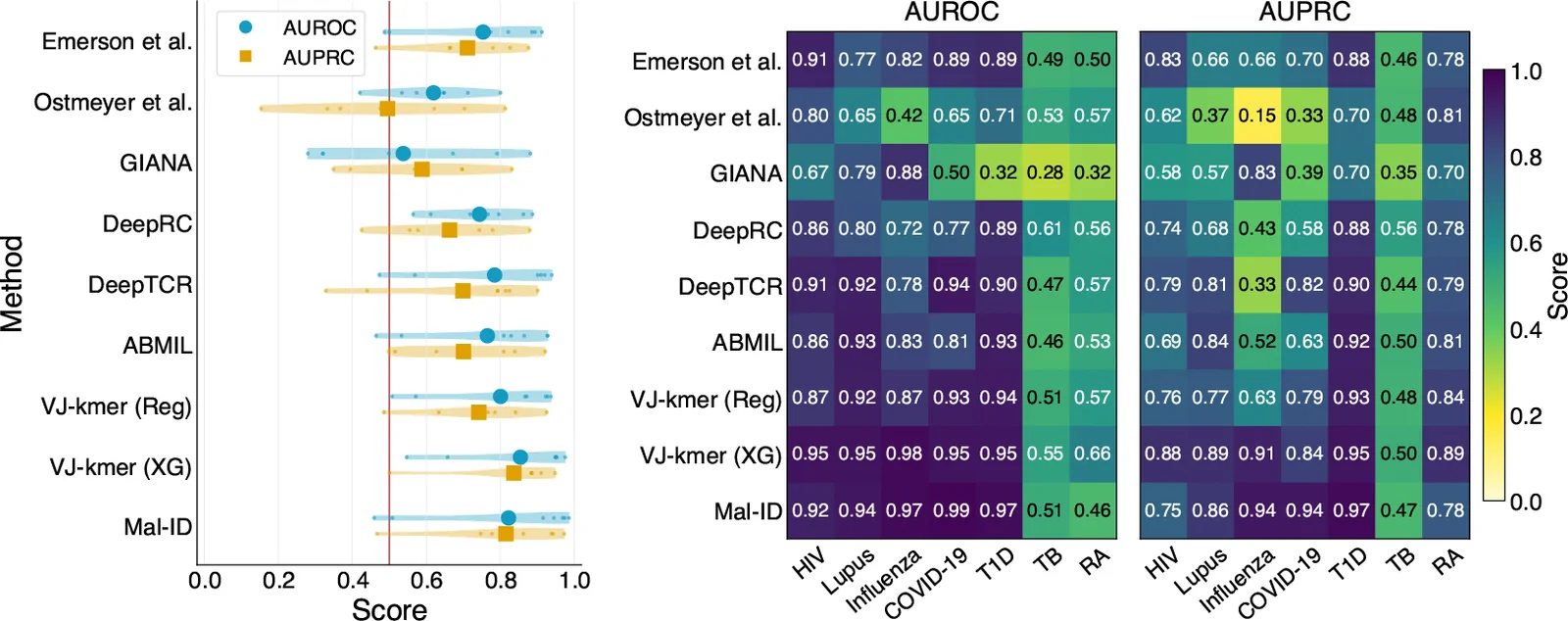
Disease classification performance across all seven diseases. (**left**) Overall method performance summarized across diseases. For each method, large markers indicate the mean AUROC or AUPRC across all seven diseases, small dots show the individual disease-specific values, and horizontal lines span the min-max range across diseases. (**right**) Heatmaps of disease-specific classification performance. Each cell reports the pooled metrics across the 3-fold cross-validation.

We first evaluated binary disease classification across all seven benchmark diseases. Figure 2 summarizes pooled out-of-fold AUROC and AUPRC values for each method and disease. Performance varied substantially across both diseases and methods. Classification by the ensemble methods Mal-ID and VJ-kmer, particularly the XGBoost variant, ranked at or near the top for most diseases under both AUROC and AUPRC. Mal-ID performed best for Influenza, COVID-19, and T1D, while VJ-kmer (XGBoost) was highly competitive across nearly all tasks and achieved the highest scores on HIV, Lupus, TB, and RA, although performance for the latter two was modest with all methods. Deep learning methods ABMIL and DeepTCR were competitive in AUROC for several diseases, but their AUPRC values were generally less consistent compared to the ensemble methods. Emerson et al. was competitive on several tasks compared to the other non-ensemble methods, particularly under AUPRC, while Ostmeyer et al. and GIANA showed more uneven disease-specific performance.

Performance differed markedly by disease. TB and RA were the most difficult classification tasks, with lower and more compressed scores across methods. Performance on the remaining datasets was higher, with several methods achieving AUROC values above 0.9. These differences indicate that classification is affected by disease context and cohort composition. The TB data in particular represent a challenging task of distinguishing patients who controlled the infection versus showed progressive disease. The RA classification was likely affected by the small healthy control sample number (20 healthy versus 74 RA samples).

### 4.2 Sequencing Depth Scaling and Computational Resources Benchmark

TCR sequencing depth can strongly affect both signal detection and predictive performance (Figure A.2). Published methods address depth variation by subsampling or normalizing counts into proportions or relative abundances, among other approaches [4, 10, 9, 15, 11]. The literature differs on whether predictive signal is driven mainly by abundance or by incidence: Ostmeyer et al. found relative motif abundance critical to performance [10], whereas Emerson et al. reported that presence/absence alone was sufficient for strong diagnostic results [4]. To test sequencing depth effects, we reran the disease-classification benchmark on datasets downsampled to common depths D ∈ {1k, 5k, 10k, 25k, 50k, 75k} unique sequences. We used data from Lupus and HIV as representative diseases with distinct classification difficulty, and tested five methods spanning distinct modeling paradigms. Additional implementation details are provided in Appendix B.4.

**Figure 3:**
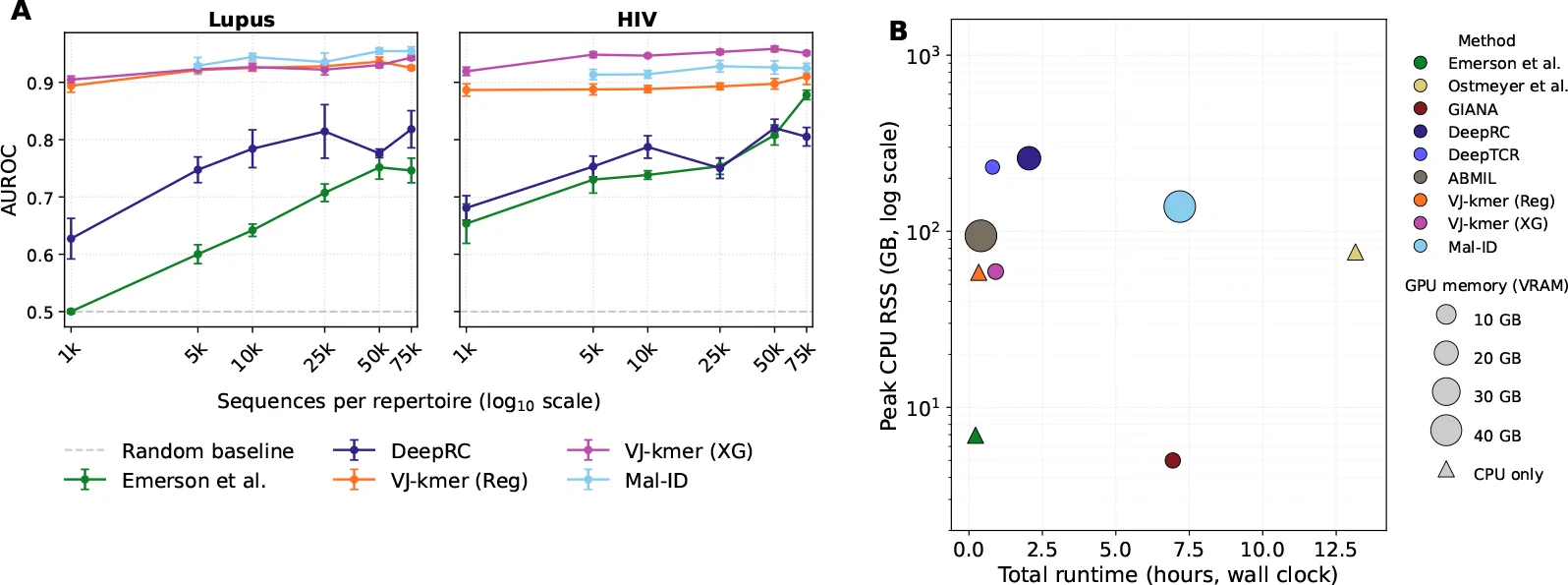
**A.** Disease classification performance as a function of sequencing depth for Lupus (**left**) and HIV (**right**). Markers show mean AUROC across five independent subsampling replicates. **B.** Computational resource benchmark of methods on the HIV dataset, measured end-to-end including data loading, training, and evaluation, and evaluated along dimensions of time (*x*-axis) and peak RAM held by the running process (*y*-axis). The size of each circular point indicates peak GPU VRAM held by the running process.

Results are shown in Figure 3A. Mal-ID and VJ-kmer (Reg and XG) were the most robust to downsampling, maintaining high AUROC even at 5k–10k sequences and showing only modest gains with additional depth. Emerson et al. showed greater dependence on sequencing depth, and DeepRC had intermediate behavior. HIV classification overall seems less sensitive to sequencing depth than Lupus. Similar trends are observed for AUPRC (Figure A.3). We observe that relatively simple models trained on intuitive featurizations of the gene identity and CDR3 information were both more robust and more performant compared to more complex deep learning and exact-match based models.

We also benchmarked time and memory resources required by each method, running each method on the HIV dataset on a single NVIDIA L40S GPU (48 GB VRAM) with 50 CPU cores allocated per run. A single run encompassed end-to-end data loading, method training, and evaluation for one fold. We used all available repertoires at each method’s default depth-filtering setting, recording wall-clock time, peak CPU resident set size (RSS, i.e. the maximum physical RAM occupied by the process) and, for GPU-accelerated methods, the peak GPU memory (VRAM) allocation during training and inference (Figure 3B). Methods spanned roughly an order of magnitude in runtime and several orders of magnitude in CPU memory footprint. Resource benchmarking details are provided in Appendix B.5.

### 4.3 Driver Sequence Identification

Specimen-level classification performance does not by itself indicate whether a method is prioritizing biologically meaningful sequences rather than broader repertoire-level correlates of disease status. We define a *driver sequence identification* task that evaluates whether methods capable of producing sequence-level scores rank known antigen-specific TCRs highly. This analysis requires per-sequence scoring functionality as implemented in Emerson et al., GIANA, DeepRC, ABMIL, and the two VJ-kmer variants. We assembled publicly available ground-truth sets of antigen-specific TCR*β* CDR3 sequences for HIV (1,747), COVID-19 (18,082), and Influenza (36,453), using VDJdb for all three diseases [19] and additionally Minervina et al. for COVID-19 [8]. Additional curation details are provided in Section B.6.

We label *drivers* as all specimen CDR3 amino-acid sequences with at least 90% Levenshtein similarity to any reference CDR3 for that disease. Within each disease-classification fold, we then use each method’s sequence-level scores to rank all CDR3s in the held-out test specimens. For each disease-positive test specimen, recall@*k* is computed as the number of labeled driver sequences among its top-*k* ranked sequences divided by the total number of labeled driver sequences in that specimen. We report specimen-level recall@*k* macro-averaged across disease-positive test repertoires for *k* ∈ {100, 1000, 10000}.

**Figure 4:**
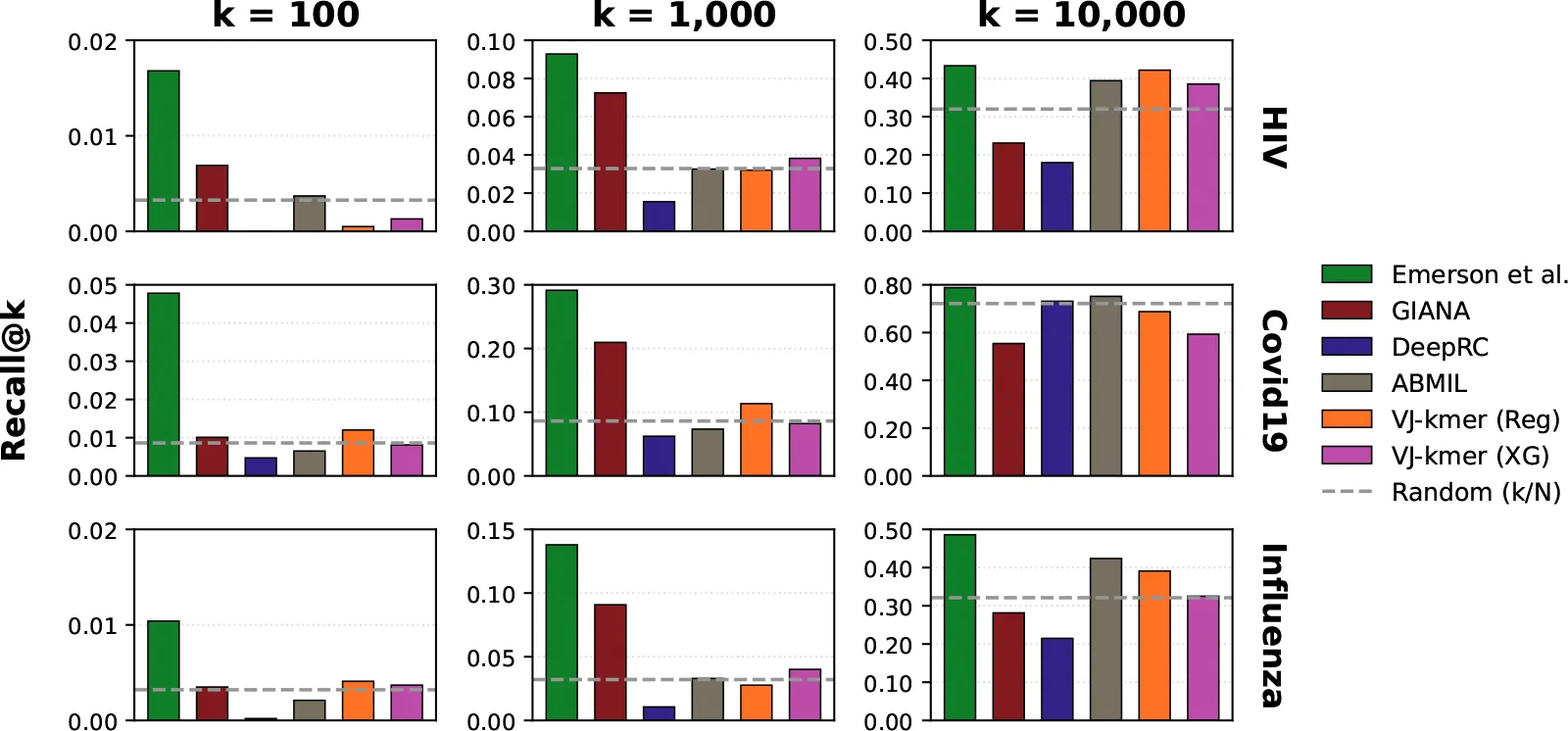
Macro-averaged specimen-level recall@*k* for *k* ∈ {100, 1000, 10000} on HIV, COVID-19, and Influenza. For each disease-positive test specimen, sequences are ranked by the method’s per-sequence score, and recall@*k* is computed as the fraction of labeled driver sequences in that specimen that appear among its top-*k* ranked sequences. Dashed gray lines indicate the random baseline, estimated by repeatedly sampling *k* sequences uniformly at random from each disease-positive repertoire, computing driver sequences recall@*k* for each random sample, and averaging over 10,000 repetitions per repertoire and then across repertoires.

Emerson et al. most consistently ranks driver sequences highly across all three diseases (Figure 4). Its advantage is most pronounced at small and intermediate *k*: at *k*=1000, Emerson et al. is clearly above the random baseline for all three diseases, whereas most other methods remain close to random or lower. GIANA performs relatively well at small *k* values for all diseases at *k*=1000 and shows some enrichment at *k*=100 for HIV; however, it performs worse than random at *k*=10000. At *k*=10000 the gap between methods narrows, with Emerson et al., ABMIL and VJ-kmer recovering a larger fraction of driver sequences than random for HIV and Influenza. For COVID-19, all methods perform close to random or worse. Absolute recall is highest for COVID-19 across all methods, consistent with the size and breadth of each disease’s VDJdb-derived ground-truth set.

### 4.4 Demographic Confounding Analysis

TCR repertoire V- and J-gene usage and CDR3 sequence patterns vary not only with disease state but also demographics and genetic background, likely due to inherited differences in immune-related genes including human leukocyte antigen (HLA) genes, which shape TCR repertoires during T-cell selection processes [9, 4, 10, 23, 24]. Age also affects repertoire diversity and composition in ways that can overlap with disease-associated signatures [9, 4, 23]. Sex can influence diseases such as lupus. Disease-associated and demographic signals may be partly entangled due to cohort selection or inherent disease biology, making it important to determine whether a classifier is learning disease biology or confounded demographic features. For example, in the data from Zaslavsky et al. [9], the Lupus cohort is predominantly female (81%), whereas the HIV cohort is predominantly of self-reported African ancestry (89%). A central question in repertoire classification studies is therefore how much of the apparent predictive signal reflects disease-associated immune structure versus demographic imbalance in the underlying data (Figure A.1).

We probe this question with two complementary analyses. First, *demographic-matched disease classification* reruns each disease-classification task after replacing the random pool of healthy controls with a subset matched to a dominant demographic confounder of the disease cohort: age for Lupus, Influenza, and COVID-19; and self-reported African ancestry for HIV. Although the Lupus cohort is strongly sex-skewed, we match on age to vary only one demographic axis at a time when constructing matched controls, noting that the female-skewed distribution reflects real-world Lupus epidemiology. For these analyses, we compare performance on equal numbers of matched-control samples and random non-demographically-matched samples, averaged over five independent draws. Second, *demographic-aware disease classification* appends demographic variables to each method’s repertoire-level feature representation, allowing us to test whether demographic information is partially redundant with repertoire-derived signal or instead adds complementary predictive power. Details on the experiment setup are in Section B.8.

**Figure 5:**
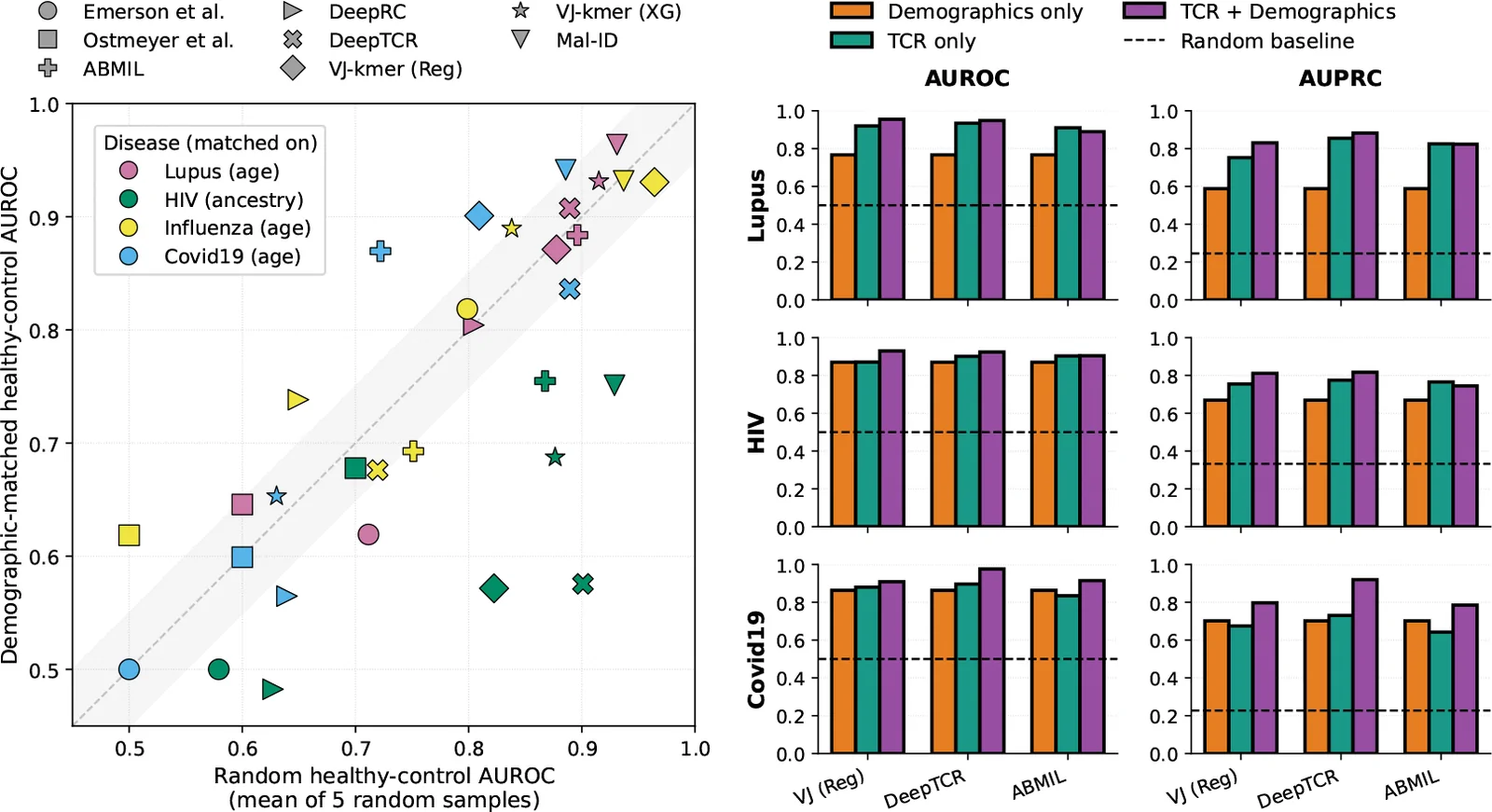
(**left**) Per-(method, disease) AUROC with random healthy controls (*x*-axis, mean of five draws) versus controls matched on the cohort’s dominant confounder (*y*-axis). Point color indicates the disease and matching variable; marker shape indicates the method. Points outside the ±0*.*05 AUROC band (gray) indicate that matching materially changed performance. (**right**) AUROC and AUPRC (3-fold CV) for three representative methods fit without (light blue) versus with (dark blue) demographic features appended to the repertoire-derived features. The annotated numbers describe the difference in metric value between the bars. The red dashed line shows the performance of a demographics-only logistic-regression baseline on the same specimens.

Figure 5 (left) reports the classification AUROC for demographically-matched and -unmatched samples. Many points lie close to the diagonal and within the gray band corresponding to an absolute AUROC difference of at most 0.05, suggesting that demographic matching usually has a modest effect on performance. For HIV, matching on ancestry frequently reduces AUROC, consistent with the strong correlation between self-reported African ancestry and HIV status in this cohort. The age-matched diseases show a more mixed pattern.

Figure 5 (right) shows the *demographic-aware disease classification* for three methods on Lupus, HIV, and COVID-19. Specimens missing any demographic field are excluded within each panel so that the comparisons are made on an identical specimen set. Demographic data for age, sex, and ancestry were not sufficiently complete for the Influenza cohort. The demographics-only baseline (red dashed line) is well above the random baseline for all three diseases, indicating that demographic variables carry substantial predictive signal, likely due to cohort imbalance. For Lupus, the repertoire-based models remain clearly above the demographics-only baseline in both AUROC and AUPRC, suggesting that demographics do not account for most of the observed signal. For HIV, this advantage is much smaller in AUROC, though it remains more apparent in AUPRC, consistent with the greater confounding of disease state and demographics in this cohort. For COVID-19, the demographics-only classification performance was comparable to several repertoire-only models, but adding repertoire features further increased performance.

## 5 Discussion

In this study, we develop the BenchRep-T framework to evaluate TCR repertoire classification approaches on a standardized dataset to enable nuanced method comparison. On the disease-classification task, we observe that the VJ-kmer methods - ensemble repertoire-level models based on V/J gene composition and gapped *k*-mer features - are highly competitive with, and in several settings outperform, more complex methods that use deep learning representations. Second, no single method dominates uniformly across all diseases: some methods are comparatively stable across tasks, whereas others show pronounced strengths and weaknesses depending on the dataset. The relative ranking of methods also depends on available computational resources, as ensemble approaches like VJ-kmer offer a favorable accuracy-to-cost tradeoff compared to deep learning methods such as DeepRC and ABMIL, which require substantially greater compute to achieve competitive performance. This heterogeneity highlights the importance of comparing methods across multiple disease settings and computational constraints, and being cautious with conclusions drawn from any single cohort. Repertoire-level performance and per-sequence interpretability also appear to capture distinct properties: methods with strong classification performance did not necessarily maximize ranking of known antigen-specific TCRs near the top of their per-sequence scores, and the statistical enrichment approach of Emerson et al. [4] recovered VDJdb-derived driver sequences more effectively than several higher-AUROC methods.

Notably, sequencing depth-scaling and demographic analyses reveal patterns that are not visible from classification performance alone. Under aggressive downsampling, the ensembles (VJ-kmer, Mal-ID) were comparatively robust, whereas the representative deep learning and statistical enrichment methods showed greater depth dependency. A plausible explanation is that V/J gene-usage composition is relatively stable under subsampling and, on its own, already carries informative disease signal, so methods that make use of these features remain effective with smaller datasets. The demographic analysis indicated that when participant demographics are strongly correlated with case/control status in the source cohort (e.g. self-reported African ancestry in the HIV dataset), classification performance can decrease when controls are matched on that confounder. A model trained on age, sex, and ancestry alone reaches non-trivial performance on several datasets, most notably HIV and COVID-19, at levels comparable to some repertoire-only predictors. This suggests that demographic structure, including both genuine repertoire variation and cohort imbalance correlated with label, can contribute to classification signal in these settings.

Several limitations should be kept in mind when interpreting these results. BenchRep-T is constrained by the scale and composition of currently available TCR datasets: our analysis was based on fewer than one thousand specimens across eight disease cohorts, some of which show notable demographic confounding with disease category (Figure A.1). Within these constraints, we examined demographic structure as carefully as the available data allowed, using both matched-control analyses and ablations that incorporate demographic covariates alongside repertoire-derived features. We note that the cohorts introduced in Zaslavsky et al. [9] contribute four of the seven disease classification tasks evaluated in BenchRep-T (HIV, Lupus, Influenza, and COVID-19). Because Mal-ID was originally developed and validated on these specimens, its performance for these diseases may reflect an advantage that is not available to the other benchmarked methods. Future benchmarks that incorporate independently collected cohorts for these diseases would help disentangle method quality from dataset familiarity.

For the T1D disease represented in two cohorts, we choose to report pooled cross-validated performance rather than strict held-out-source generalization (i.e. zero-shot transfer). Since the two cohorts were generated with different sequencing library preparation protocols, zero-shot transfer without explicit batch or technical correction causes batch effects and disease status to become confounded. We note that domain-adaptation strategies for cross-platform generalization are an important direction for future work. Another limitation is that we evaluate repertoire classification only in binary settings, rather than as a single multiclass diagnostic task. This was partly a practical choice, since among benchmarked methods only some support multiclass classification, while the others are formulated for one-disease-versus-control problems. Consequently, strong performance in our benchmark does not necessarily imply equally strong performance in a multiclass setting, where methods must separate disease-specific signal from both healthy background and competing disease classes.

Looking forward, larger and more demographically balanced cohorts with matched control participants, together with principled strategies for handling cross-cohort heterogeneity, should make it possible to evaluate these questions more directly and under more realistic deployment conditions. Larger and improved datasets would also enable the development of more powerful machine learning methods for TCR repertoire classification. We release BenchRep-T as an extensible framework and set of datasets to support that work and to encourage more rigorous and reproducible progress in immune repertoire-based disease diagnostics.

## Supplementary Material

Supplement 1

## Data Availability

The code and documentation for our data processing and evaluations is available at https://github.com/boyd-lab/BenchRep-T. The Mal-ID re-implementation is available at https://github.com/liel-cohen/Mal-ID-Lite. The raw repertoire files from the Mal-ID dataset used in the evaluations is available at https://www.synapse.org/Synapse:syn61987835/wiki/629312. The raw repertoire files from the immunoSEQ dataset are available at https://clients.adaptivebiotech.com/pub/[postfix] with the following postfixes: mitchell-2022-jcii, musvosvi-2022-nm, and mustjoki-2017-natcomms.
