# Supplementary material for: BenchRep-T: A Systematic Evaluation of T-Cell Repertoire-Based Disease Diagnostics
Source: bioRxiv. 2026 Jun 10:2026.06.09.727013. Preprint. [Version 1] doi: 10.64898/2026.06.09.727013 (PMC13277846; doi:10.64898/2026.06.09.727013)
Supplement: Supplementary file 1 [file NIHPP2026.06.09.727013v1-supplement-1.pdf]

bioRxiv preprint doi: https://doi.org/10.64898/2026.06.09.727013; this version posted June 10, 2026. The copyright holder for this preprint (which was not certified by peer review) is the author/funder, who has granted bioRxiv a license to display the preprint in perpetuity. It is made available under a CC-BY 4.0 International license.

A Supplementary Figures and Tables

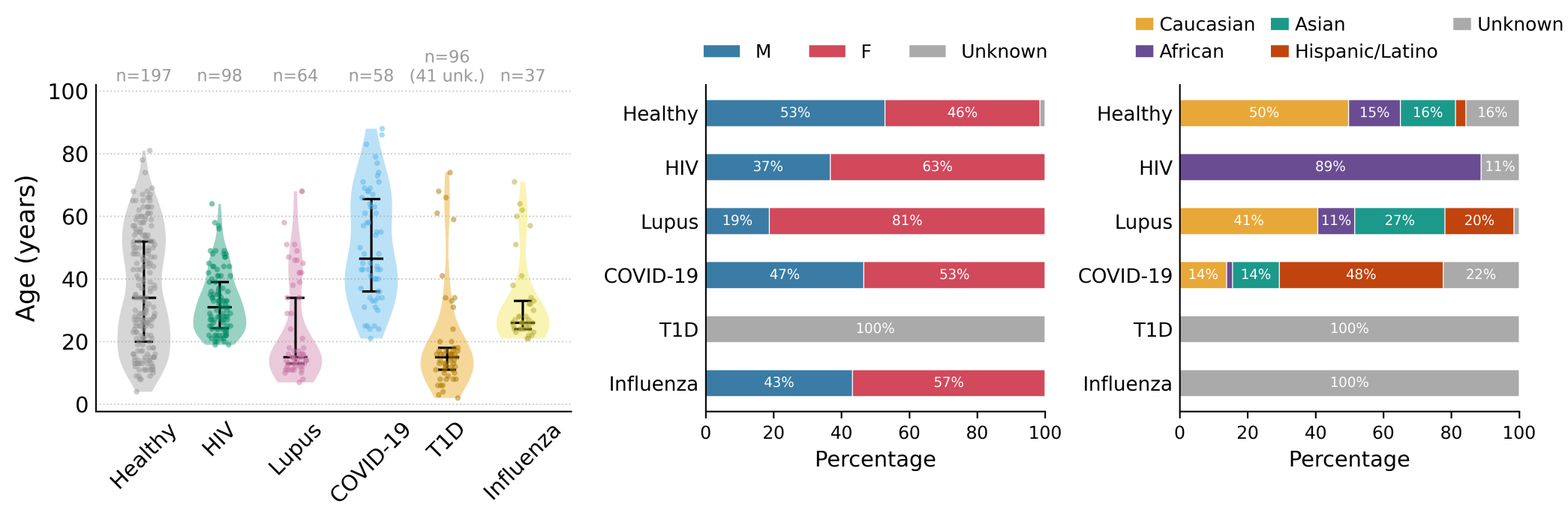

Figure A.1: Demographics distribution of the Mal-ID Cohort across age, sex, and ancestry.

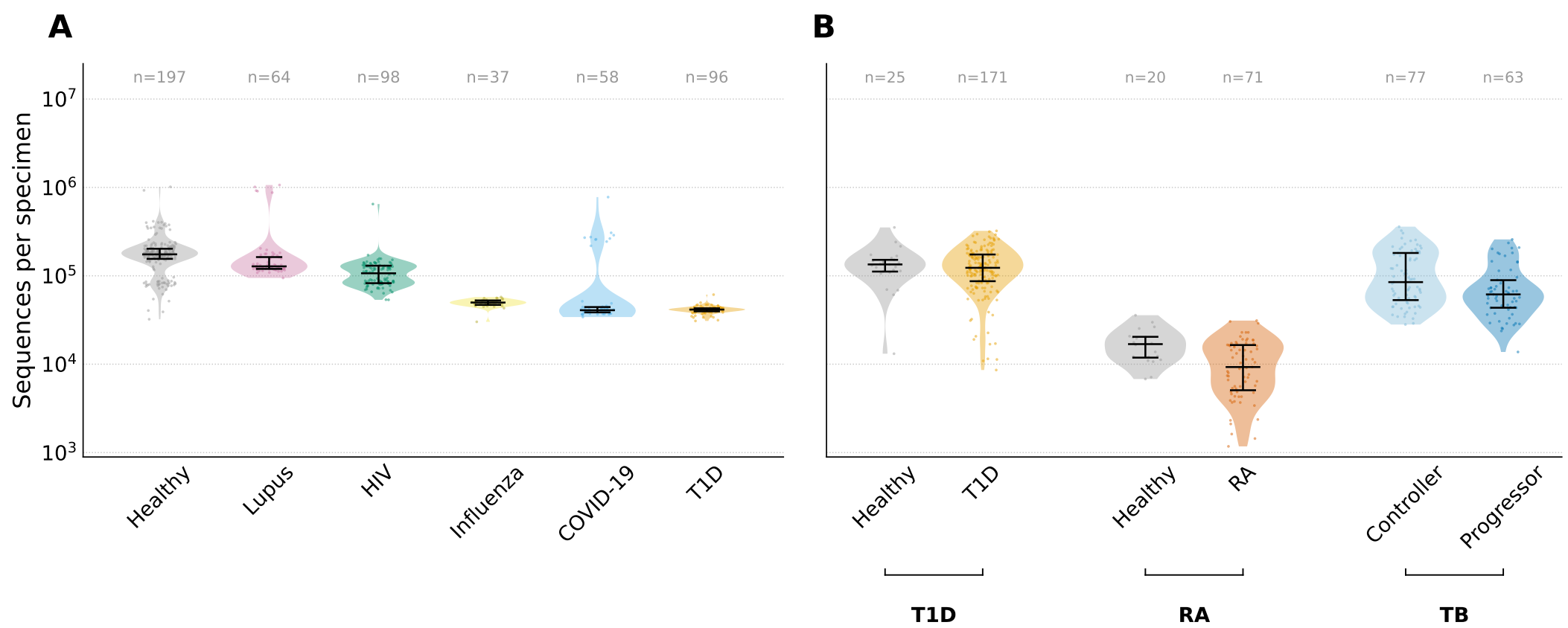

Figure A.2: Repertoire sequencing depth distribution of the Mal-ID Cohort and the immunoSEQ datasets.

bioRxiv preprint doi: https://doi.org/10.64898/2026.06.09.727013; this version posted June 10, 2026. The copyright holder for this preprint (which was not certified by peer review) is the author/funder, who has granted bioRxiv a license to display the preprint in perpetuity. It is made available under a CC-BY 4.0 International license.

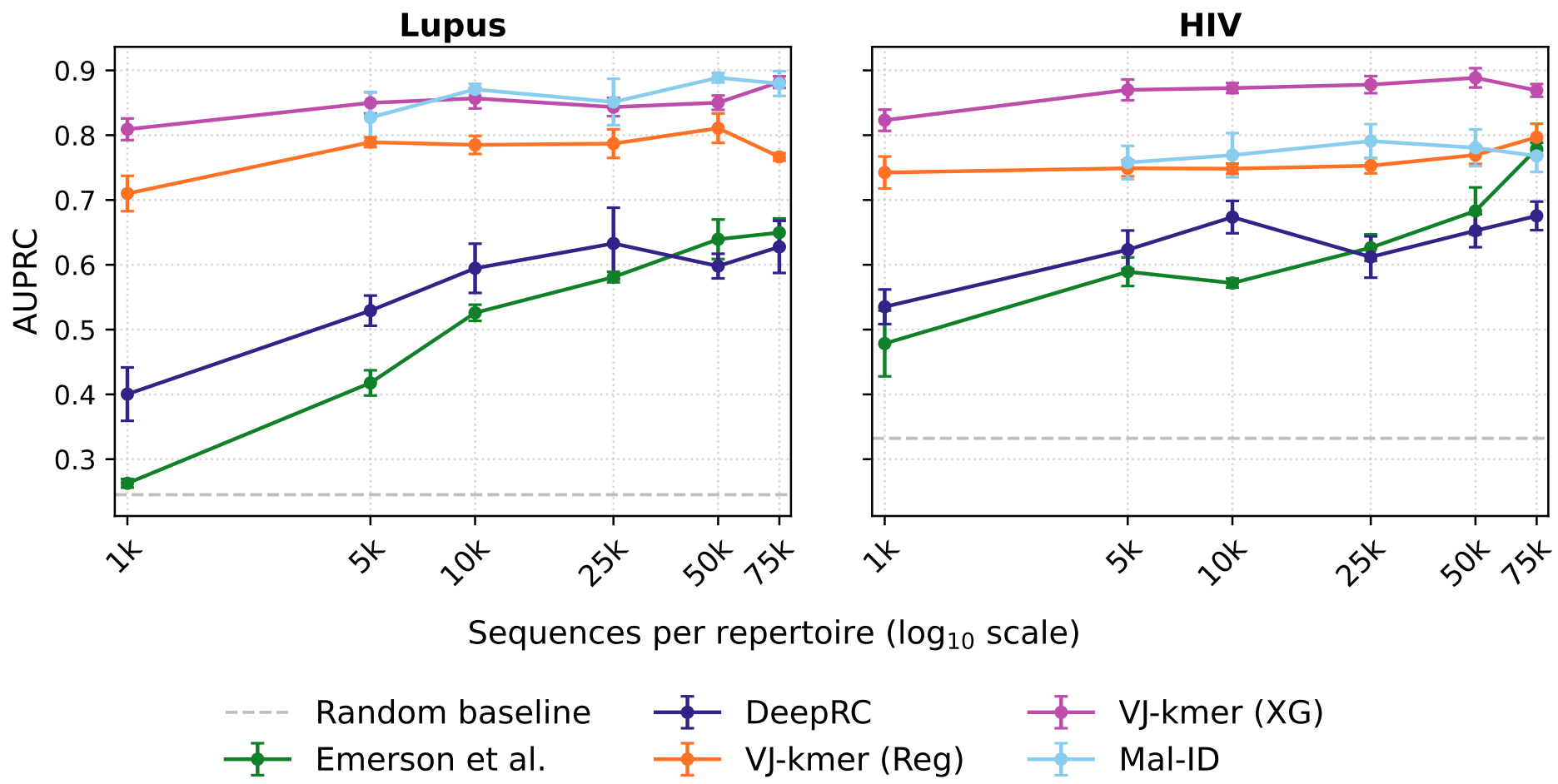

Figure A.3: Disease-classification performance as a function of sequencing depth for Lupus (**left**) and HIV (**right**). Markers show mean AUPRC across five independent subsampling replicates, with error bars denoting the standard error across replicates.

Accuracy

Disease	M2-abstained*	M2-fill-0.5	M2-fill-mean-M1-M3	Abstention	Test Abstained	Train Abstained
Covid19	0.9412	0.9451	0.9451	0.40%	1	0
HIV	0.8475	0.8678	0.8678	2.00%	6	0
Influenza	0.8761	0.9444	0.9444	7.30%	17	7
Lupus	0.8276	0.8851	0.8851	5.00%	13	5
T1D	0.9215	0.9386	0.9386	2.70%	8	1

*Abstained samples counted as erroneous predictions

AUROC

Disease	M2-abstained*	M2-fill-0.5	M2-fill-mean-M1-M3	Abstention	Test Abstained	Train Abstained
Covid19	0.9859	0.9857	0.9858	0.40%	1	0
HIV	0.9131	0.9155	0.916	2.00%	6	0
Influenza	0.9751	0.9691	0.9745	7.30%	17	7
Lupus	0.9159	0.9446	0.945	5.00%	13	5
T1D	0.9819	0.966	0.9633	2.70%	8	1

*Abstained samples not included in metric computation

AUPRC Comparison

Disease	M2-abstained*	M2-fill-0.5	M2-fill-mean-M1-M3	Abstention	Test Abstained	Train Abstained
Covid19	0.9386	0.9384	0.9387	0.40%	1	0
HIV	0.7308	0.7442	0.747	2.00%	6	0
Influenza	0.9535	0.919	0.9427	7.30%	17	7
Lupus	0.791	0.863	0.8626	5.00%	13	5
T1D	0.9551	0.9403	0.9383	2.70%	8	1

*Abstained samples not included in metric computation

Figure A.4: Handling model-2 abstentions in the Mal-ID ensemble. We compare the original abstention-based ensemble to two strategies for imputing missing model-2 outputs on abstained specimens: replacing the missing value with 0.5, or replacing it with the per-specimen mean of that specimen's model-1 and model-3 predictions. We report AUROC and AUPRC across diseases, together with abstention rates and the numbers of abstained train and test specimens.

bioRxiv preprint doi: https://doi.org/10.64898/2026.06.09.727013; this version posted June 10, 2026. The copyright holder for this preprint (which was not certified by peer review) is the author/funder, who has granted bioRxiv a license to display the preprint in perpetuity. It is made available under a CC-BY 4.0 International license.

Table A.1: AUROC (**Top**) / AUPRC (**Bottom**) across k-mer featurizations on five disease classification tasks. We sweep k-mer length ($k \in \{3, 4, 5\}$) and gapping (ungapped vs. gapped+ungapped combined) for two model variants: the full *Ensemble* that combines k-mer and V/J-gene features, and a *k-mer-only* ablation. The *V/J only* baseline uses V/J-gene features alone and is therefore independent of the k-mer sweep. We also report the per-disease range $\Delta = \max - \min$ across the configurations. **Take-away:** the ensemble is robust to k-mer featurization choice (Δ AUROC ≤ 0.030 on every disease), whereas the k-mer-only model is highly sensitive (e.g. Lupus spans 0.746–0.839), indicating that V/J features absorb most of the variation introduced by the k-mer choice.

Model	Featurization	Lupus	T1D	HIV	Influenza	COVID-19
Ensemble (k-mer + V/J)	$k = 3$, ungapped	0.9231	0.9883	0.9093	0.9791	0.9288
	$k = 3$, gapped+ungapped	0.9231	0.9886	0.9012	0.9749	0.9299
	$k = 4$, ungapped	0.9016	0.9885	0.8936	0.9804	0.9197
	$k = 4$, gapped+ungapped	0.9065	0.9886	0.8895	0.9764	0.9264
	$k = 5$, ungapped	0.9173	0.9887	0.8796	0.9778	0.9452
	$k = 5$, gapped+ungapped	0.9083	0.9885	0.8915	0.9796	0.9445
	Range (Δ)	**0.0215**	**0.0004**	**0.0297**	**0.0055**	**0.0255**
k-mer only	$k = 3$, ungapped	0.7456	0.9897	0.8898	0.9363	0.8962
	$k = 3$, gapped+ungapped	0.7585	0.9880	0.8903	0.9459	0.8937
	$k = 4$, ungapped	0.7877	0.9849	0.8407	0.9527	0.8571
	$k = 4$, gapped+ungapped	0.8005	0.9818	0.8155	0.9525	0.8770
	$k = 5$, ungapped	0.8385	0.9867	0.8729	0.9617	0.8922
	$k = 5$, gapped+ungapped	0.8193	0.9862	0.8388	0.9660	0.8933
	Range (Δ)	0.0929	0.0079	0.0748	0.0297	0.0391
V/J only	—	0.9230	0.9885	0.8896	0.9829	0.9373

Model	Featurization	Lupus	T1D	HIV	Influenza	COVID-19
Ensemble (k-mer + V/J)	$k = 3$, ungapped	0.7283	0.9588	0.8113	0.8450	0.8113
	$k = 3$, gapped+ungapped	0.7283	0.9596	0.8088	0.8304	0.8106
	$k = 4$, ungapped	0.7375	0.9598	0.7581	0.8539	0.8001
	$k = 4$, gapped+ungapped	0.7350	0.9600	0.7511	0.8343	0.8078
	$k = 5$, ungapped	0.7591	0.9604	0.7236	0.8562	0.8264
	$k = 5$, gapped+ungapped	0.7478	0.9598	0.7479	0.8473	0.8247
	Range (Δ)	**0.0308**	**0.0016**	**0.0877**	**0.0258**	**0.0263**
k-mer only	$k = 3$, ungapped	0.4375	0.9642	0.8155	0.5991	0.7732
	$k = 3$, gapped+ungapped	0.4660	0.9611	0.8224	0.6425	0.7572
	$k = 4$, ungapped	0.6705	0.9589	0.6806	0.6997	0.7283
	$k = 4$, gapped+ungapped	0.6880	0.9360	0.6508	0.6863	0.7640
	$k = 5$, ungapped	0.7781	0.9510	0.7321	0.7552	0.7836
	$k = 5$, gapped+ungapped	0.7602	0.9575	0.6514	0.7682	0.7505
	Range (Δ)	0.3406	0.0282	0.1716	0.1691	0.0553
V/J only	—	0.7283	0.9593	0.7216	0.8722	0.8529

bioRxiv preprint doi: https://doi.org/10.64898/2026.06.09.727013; this version posted June 10, 2026. The copyright holder for this preprint (which was not certified by peer review) is the author/funder, who has granted bioRxiv a license to display the preprint in perpetuity. It is made available under a CC-BY 4.0 International license.

**Component**	**CLI argument**	**Value**	**Notes**
Model 1	`-model1-n-pcs`	`15`	
Model 1	`-model1-l1-ratio`	`per-locus`	automatically sets `1.0` (lasso) for TCR
Model 1	`-model1-model-name`	`lasso_cv`	
Model 2	`-model2-sequence-identity-threshold`	`per-locus`	automatically sets `0.90` for TCR
Model 2	`-model2-p-values`	`[0.0005, 0.001, 0.005, 0.01, 0.05]`	
Model 2	`-model2-retrain-on-full-train`	`False`	
Model 2	`-model2-abstention-strategy`	`fill_models13_mean`	
Model 3	`-model3-aggregation-strategy`	`entropy_percentile_cutoff`	
Model 3	`-model3-entropy-bottom-percentile`	`0.01`	
Model 3	`-model3-tuning-cv-splits`	`3`	
Model 3	`-model3-n-estimators-stage2`	`100`	
Ensemble	`-model2-abstention-strategy`	`fill_models13_mean`	
Caching	`-clone-id-use-aa`		immunoSEQ datasets only
Embeddings	`-batch-size`	`15,000`	

Table A.2: CLI arguments used for the Mal-ID runs.

bioRxiv preprint doi: https://doi.org/10.64898/2026.06.09.727013; this version posted June 10, 2026. The copyright holder for this preprint (which was not certified by peer review) is the author/funder, who has granted bioRxiv a license to display the preprint in perpetuity. It is made available under a CC-BY 4.0 International license.

B Technical Appendices

B.1 Dataset Preprocessing

All repertoire files consumed by the benchmarked methods are harmonized to a single AIRR-compliant schema with `cdr3_aa`, `v_call`, and `j_call` as the sequence-level columns, regardless of which source they originated from. The Mal-ID and immunoSEQ data enter the pipeline in different raw formats and therefore follow different but conceptually parallel cleanup paths, described below.

Mal-ID. The Mal-ID release is distributed in an internal, per-participant tabular format produced by an IgBLAST-based annotation pipeline. For each participant file we (i) drop non-productive rearrangements and low-confidence V assignments, (ii) strip the whitespace inserted by IgBLAST between residues in each amino-acid field and uppercase the result, (iii) drop any row with a missing CDR3, V, or J call or with a non-standard amino-acid character in its CDR3 (e.g. `*` for a stop codon, `X` for an ambiguous residue), and (iv) collapse a small number of V-gene alleles that are known to be indistinguishable under the FR3 primers used in the Mal-ID assay to a canonical allele, following Meysman et al. [23] The cleaned per-participant tables are renamed into AIRR column conventions and split by `repertoire_id` into one file per specimen, yielding the 550 specimen-level files.

immunoSEQ cohorts. The three immunoSEQ cohorts (T1D, TB, RA) are released in the Adaptive/immunoSEQ tabular format, which differs from AIRR in both column naming and gene nomenclature. We rename `aminoAcid`, `vGeneName`, and `jGeneName` to `cdr3_aa`, `v_call`, and `j_call`; harmonize V/J gene names by converting the Adaptive prefix (e.g. `TCRBV07-02`) into the AIRR/IMGT form (`TRBV7-2`), taking the first gene for ambiguous slash-separated calls, and stripping allele annotations so that the allele resolution matches Mal-ID; and trim the first and last residue of each CDR3 so that the flanking conserved cysteine and phenylalanine are removed and the CDR3 definition matches the Mal-ID files. No productivity or V-score filters are applied to the immunoSEQ cohorts because those fields are not reported in the Adaptive release. We additionally exclude any immunoSEQ specimen whose post-preprocessing repertoire contains fewer than $1{,}000$ unique sequences, as such specimens carry too little signal to be informative and are dominated by sampling noise.

Cross-source gene-label reconciliation. The Mal-ID and immunoSEQ pipelines independently produce AIRR-style V-gene labels, but the two conventions still disagree on single-member TRBV families: Mal-ID (IMGT) writes these without a trailing `-1`, while the Adaptive-derived files retain it (e.g. `TRBV13-1` vs. `TRBV13`). When an immunoSEQ cohort is merged with Mal-ID (as in the T1D evaluation of Section 4.1) we collapse the Adaptive-style `-1` suffix on the ten singleton families (`TRBV2`, `TRBV9`, `TRBV13`–`TRBV15`, `TRBV18`, `TRBV19`, `TRBV27`, `TRBV28`, `TRBV30`) so that the same gene receives the same label across both sources. The collapse is applied only when sources are merged. Within-source evaluations preserve each source's native labels.

B.2 Methods Details

B.2.1 Statistical Disease-Signature Methods

Emerson et al. (2017) The immunosequencing model of Emerson et al. [4] identifies individual TCRβ clones that are differentially present in cases versus controls and uses their occurrence counts as a generative signal. For each unique (V gene, J gene, CDR3 amino-acid sequence) triplet observed in at least two training subjects, we perform a one-sided Fisher's exact test on the 2×2 contingency table of presence/absence against disease label. Triplets with p-value less than 10^{-4} form the diagnostic set $\mathcal{T}$. For each subject i we compute the repertoire size n_i (number of unique triplets in the subject) and the diagnostic burden k_i (number of those triplets that fall in $\mathcal{T}$), and fit class-conditional Beta-Binomial distributions $\mathrm{BB}(k_i \mid n_i, \alpha_c, \beta_c)$ for $c \in \{\text{disease}, \text{healthy}\}$ on the negative log-likelihood. At test time we compute log-posterior odds with a Laplace-smoothed class prior $(N_c + 1)/(N + 2)$. The model exposes a single core hyperparameter, which is the p-value threshold (default 1e-4, following the paper's cross-validation–optimized value), which sets the Fisher's exact test cutoff used to select disease-associated diagnostic TCRs.

Ostmeyer et al. (2019) Ostmeyer et al. [10] models a repertoire as a bag of 4-mer motifs. After stripping the first three and last three residues of each CDR3, every overlapping 4-mer is encoded as

bioRxiv preprint doi: https://doi.org/10.64898/2026.06.09.727013; this version posted June 10, 2026. The copyright holder for this preprint (which was not certified by peer review) is the author/funder, who has granted bioRxiv a license to display the preprint in perpetuity. It is made available under a CC-BY 4.0 International license.

a 20-dimensional vector by concatenating the five Atchley factors [25] of its residues, augmented with the log of its clone-size-weighted relative abundance to give a 21-dimensional feature. A logistic regression is trained on these bags under a max-aggregation MIL objective, so each bag's gradient flows only through its highest-scoring "witness" 4-mer. We follow the authors' prescription of repeated solver restarts with Gaussian weight initialization and no L1/L2 regularization, keeping the restart with the lowest training loss. Optimization is controlled by two parameters: number of Gaussian restarts, the number of random L-BFGS-B initializations from which the lowest-training-loss solution is retained (the original paper used 250,000 restarts; our default, 200, trades compute for landscape coverage), and max number of iteration for the lbfgsb solver (default 1000), a per-restart cap on L-BFGS-B iterations that serves as a safety bound since runs typically converge within 50–200 gradient evaluations.

B.2.2 Ensemble of Engineered Repertoire-level Representations

V/J gene + Gapped k-mer (VJ-kmer) ensemble: We represent each repertoire using two simple feature dictionaries of relative frequencies: (i) V-gene and J-gene usage, each normalized within its own gene type, and (ii) all CDR3 4-mers plus their four single-position gapped variants (“`X_YZ`”, etc.), normalized by the total k-mer count. Each feature set is passed to an L_1-penalized logistic regression; the inverse-regularization strength C is selected independently for each sub-model by 5-fold stratified CV from `[1.0, 0.2, 0.1, 0.05, 0.03]` using AUROC. The two sub-model probabilities are combined as $p = \alpha \cdot p_{\text{kmer}} + (1-\alpha) \cdot p_{\text{VJ}}$, with $\alpha \in \{0.0, 0.1, \ldots, 1.0\}$ tuned on an internal $80/20$ validation split. We report the performance of two sub-models (with k-mer features only and V/J gene features only) in isolation as ablations (Table A.1).

We additionally evaluate an XGBoost [26] variant that swaps each L_1-penalized logistic regression base learner for a gradient-boosted tree classifier, keeping the same feature dictionaries and the same α sweep. Here we use `binary:logistic` objective with up to 1,000 boosting rounds and early stopping after 20 rounds without improvement on a validation set. Hyperparameter tuning is conducted through a two-stage deterministic grid search:

1. Stage 1 sweeps `max_depth` $\in \{3, 4, 5, 6\} \times$ `learning_rate` $\in \{0.01, 0.03, 0.05, 0.1\}$ (16 combinations).
2. Stage 2 fixes the best Stage 1 values, then sweeps `subsample` $\in \{0.7, 0.8, 1.0\} \times$ `colsample_bytree` $\in \{0.7, 0.8, 1.0\} \times$ `min_child_weight` $\in \{1, 3, 5\}$ (27 combinations).

Mal-ID (2025) Mal-ID (MAchine Learning for Immunological Diagnosis) [9] is an ensemble framework that combines three complementary repertoire representations for the BCR and TCR repertoires separately. These include (i) a repertoire-composition model based on normalized V/J gene usage and, for BCR, somatic hypermutation features; (ii) a convergent-clustering model that groups sequences by V gene, J gene, and CDR3 length, clusters sequences within each group by CDR3 amino acid similarity, identifies disease-enriched clusters by statistical testing, and trains a classifier on cluster-count features; and (iii) a protein language model pipeline that embeds individual CDR3 sequences with ESM-2 [21], scores them with sequence-level classifiers specialized by V gene, and aggregates these predictions to the specimen level with a second-stage classifier. Further details regarding adaptation applied in this study are in Appendix B.3. The Mal-ID re-implementation is available at `https://github.com/liel-cohen/Mal-ID-Lite`.

Mal-ID-Lite arguments used for the study runs are listed in Table A.2.

B.2.3 Clustering-based Methods

GIANA, Liu et al. (2021) GIANA [11] uses a precomputed substitution-matrix embedding, derived from BLOSUM-based distances and multi-dimensional scaling, to encode each CDR3 into a 96-dimensional isometric vector. Training sequences are clustered with a length-adjusted isometric-distance threshold, followed by V-gene and Smith-Waterman refinement. For each reference cluster, we compute the fraction of training sequences from the target disease, excluding clusters with more than 100 reference TCRs. A test specimen's score is the mean disease fraction across its TCRs that are assigned to reference clusters. TCRs without a reference cluster assignment are omitted from the mean, and specimens with no assigned TCRs receive score zero. Up to 10,000 sequences per

bioRxiv preprint doi: https://doi.org/10.64898/2026.06.09.727013; this version posted June 10, 2026. The copyright holder for this preprint (which was not certified by peer review) is the author/funder, who has granted bioRxiv a license to display the preprint in perpetuity. It is made available under a CC-BY 4.0 International license.

specimen are kept. We use method defaults for parameters, including isometric distance threshold 7 in exact mode, Smith-Waterman score threshold 3.3, and V-gene similarity threshold 3.7.

B.2.4 Deep Learning-based Methods

Attention-based Multiple Instance Learning (ABMIL) We implement an attention-based MIL model [22] that replaces hand-designed features with end-to-end learned embeddings. A learned amino-acid embedding (hidden dim. 64) is passed through a three-layer 1-D CNN (kernel size 5, channel dimensions $32 \rightarrow 64 \rightarrow 128$, LeakyReLU activations, dropout 0.25) with global max-pooling to produce a per-sequence vector; learned V- and J-gene embeddings (hidden dim. 48 each) are concatenated to this vector. A gated-attention aggregator pools per-sequence features into a repertoire-level representation via weighted summation, followed by a single linear layer and sigmoid classifier. The model is trained with Adam (learning rate 5×10^{-4}, weight decay 10^{-4}) to minimize binary cross-entropy with positive-class weighting ($n_{\text{neg}}/n_{\text{pos}}$), using early stopping (patience 10 epochs) on an internal 20% validation split of bags. Training runs for up to 100 epochs; each epoch subsamples at most $10{,}000$ sequences per bag.

DeepRC, Widrich et al. (2020) DeepRC [15] is a multiple-instance repertoire classifier based on modern Hopfield networks. Each CDR3 is represented by one-hot amino-acid features (20 channels) augmented with three positional features. A single-layer 1-D convolutional sequence encoder (`Conv1d` with 23 input channels, 32 kernels of size 9, SELU activation, and global max-pooling) maps each sequence to a fixed-dimensional embedding. We use the authors' PyTorch implementation with parameter defaults retained from their CNN sequence encoder and attention architecture. Training uses Adam (learning rate 10^{-4}) with L_2 weight decay to minimize binary cross-entropy over a budget of $10{,}000$ gradient updates with a batch size of 32 repertoires. Up to $10{,}000$ sequences are subsampled per repertoire during training.

DeepTCR, Sidhom et al. (2021) DeepTCR [16] is a convolutional multi-instance learning architecture for repertoire-level prediction. Each CDR3 is represented with a trainable amino-acid embedding (hidden dim. 64) passed through a 1-D convolutional layer (kernel size 5, LeakyReLU activation, dropout), while V- and J-gene identities are captured by learned gene embeddings (hidden dim. 48 each). Sequences sharing identical CDR3s are first aggregated by summing their clone counts; the model then maps each unique sequence to a learned concept space and aggregates across the repertoire via frequency-weighted summation. The model is implemented in TensorFlow and trained with Adam (lr$=10^{-3}$) to minimize softmax cross-entropy augmented with a per-sample hinge regularization term ($\max(0,\, \ell - 0.1)$) using a batch size of 25 repertoires. Early stopping monitors validation loss over a rolling window of 10 epochs and halts training when relative improvement falls below a threshold of 0.25, subject to a minimum of 25 epochs.

B.3 Mal-ID Adaptations

In the original formulation, the three BCR and three TCR base models are combined by a logistic-regression metamodel to predict immune status. Mal-ID was introduced as a multiclass framework operating jointly on BCR and TCR repertoires, with the strongest performance obtained by combining both cell types and all three model components. To integrate Mal-ID into BenchRep-T, we implemented a streamlined reimplementation of the published pipeline, which preserves the model of the original method while making it easier to run consistently across benchmark tasks, folds, and future reuse settings. Two adjustments were introduced for the BenchRep-T benchmark setting.

Adaptive entropy threshold in model 3. In the published Mal-ID configuration, model 3 aggregates sequence-level class probabilities after filtering out high-entropy sequences, so that specimen-level features are computed from lower-entropy, more class-informative predictions. In our benchmark setting, we found that using a fixed entropy cutoff could, in some folds, filter all sequences from a specimen or even nearly all sequences in the training data for a given model configuration, leading to degenerate post-aggregation features. To avoid this behavior while preserving the intended preference for lower-entropy sequences, we replaced the fixed cutoff with a training-set-based percentile threshold (0.01%). For each training fold, we estimate the entropy distribution of sequence-level predictions on the training data and store the corresponding 0.01th-percentile value as part of the fitted model. This learned cutoff is then applied unchanged at inference time to any validation

bioRxiv preprint doi: https://doi.org/10.64898/2026.06.09.727013; this version posted June 10, 2026. The copyright holder for this preprint (which was not certified by peer review) is the author/funder, who has granted bioRxiv a license to display the preprint in perpetuity. It is made available under a CC-BY 4.0 International license.

or test repertoire scored by that model. The result is a filter that adapts to the training-time score distribution while avoiding collapse of the post-filtering sequence set.

Replacing model-2 abstentions in the ensemble. In the original Mal-ID pipeline, model 2 abstains when no sequence in a specimen matches any disease-enriched cluster, and these abstentions propagate to the ensemble model, which abstains from them as well. In BenchRep-T, however, such abstentions would make AUROC and AUPRC comparisons across methods difficult, because different methods would effectively be evaluated on different subsets of specimens. We therefore replaced missing model-2 outputs with filled values in both the ensemble training and test matrices.

We compared three strategies: retaining abstentions, filling model 2 abstained sample predictions with a constant value of 0.5, and filling with the mean of the corresponding model-1 and model-3 predictions. As shown in Figure A.4, the two filling strategies produced very similar results overall, and the model-1/model-3 mean fill was slightly better on average. Relative to retaining abstentions, filling had little effect on most diseases, improved performance in some settings, and enabled every specimen to receive an ensemble prediction. Performance on the subset of non-abstained specimens remained broadly similar, indicating that the fill strategy did not materially distort ensemble behavior on samples that were already scoreable. We therefore use the model-1/model-3 mean fill strategy throughout BenchRep-T.

B.4 Sequencing-depth scaling protocol

For the sequencing-depth scaling analysis, we downsample each repertoire in both the training and test sets to a common target depth $D \in \{1\text{k}, 5\text{k}, 10\text{k}, 25\text{k}, 50\text{k}, 75\text{k}\}$ unique sequences. To isolate the effect of depth from resampling noise, downsampling indices are pre-generated once per repertoire with a fixed seed and nested across depths: the D sequences used at depth D are always a subset of those used at any larger depth. We generate five independent downsampling replicates per depth and report mean AUROC with standard error across replicates. Methods that impose a default maximum sequencing-depth filter were overridden to accept the target depth D, ensuring that all methods are evaluated on the same downsampled repertoires at every depth.

The experiment is run on Lupus and HIV, chosen as representative diseases with distinct classification difficulty in the Mal-ID benchmark, and on five methods spanning distinct modeling paradigms: Emerson et al., DeepRC, VJ-kmer (Reg), VJ-kmer (XG), and Mal-ID. Specimens with fewer than 75k sequences are excluded so that all methods are compared on an identical specimen set across depths.

B.5 Computational resource benchmarking

Computational resource usage was measured by running each method on a single NVIDIA L40S GPU (48 GB VRAM) with 50 CPU cores allocated per run. Computational benchmarking was run on the HIV dataset, encompassing data loading, featurization, model training, cross-validation, and evaluation. Wall-clock time was recorded using Python's `time.perf_counter()` from process launch to termination. Peak CPU memory was estimated as the maximum resident set size (RSS), sampled from `/proc/<pid>/status` across the full process tree (including child processes) at 0.5-second intervals throughout execution. For GPU-accelerated methods, peak GPU memory was similarly polled at 0.5-second intervals via `nvidia-smi`, and a memory delta was computed by subtracting the pre-run GPU baseline to isolate method-specific allocation from ambient device usage. Each method was launched in an isolated process group, and a background sampling thread recorded peak values continuously until process exit. Total wall-clock time, peak RAM usage estimated as RSS, and peak GPU VRAM usage were recorded for each method.

B.6 Ground Truth Driver Sequence Extraction

For each of HIV, COVID-19, and Influenza, we retained VDJdb entries [19] with confidence score ≥ 2, yielding 1,747 HIV, 2,355 COVID-19, and 36,453 Influenza reference CDR3s after deduplication within VDJdb. For COVID-19, we further augmented the reference set with 15,727 experimentally validated SARS-CoV-2-specific TCRβ clonotypes from Minervina et al. [8], obtained using single-cell assays and retained only for specificity-confirmed binders. After deduplication against VDJdb, the final COVID-19 reference set contained 18,082 unique CDR3s.

bioRxiv preprint doi: https://doi.org/10.64898/2026.06.09.727013; this version posted June 10, 2026. The copyright holder for this preprint (which was not certified by peer review) is the author/funder, who has granted bioRxiv a license to display the preprint in perpetuity. It is made available under a CC-BY 4.0 International license.

B.7 Driver sequence identification protocol

For models that support sequence-level ranking (either as prescribed by their authors or with minimal modifications), driver sequence identification was evaluated by using each trained disease classification model to assign a disease-association score to individual CDR3 sequences within held-out repertoires. For each repertoire, CDR3s were ranked by this score and compared against the ground-truth disease-associated CDR3 sequences present in each repertoire, as determined by the sequence extraction procedure described above.

Emerson et al. We used the Fisher's exact test statistics computed from training repertoires as sequence-level scores. Within each cross-validation fold, the p-value threshold was first selected on an internal train/validation split using validation AUROC, and TCR statistics were then recomputed on all non-test repertoires. Each test repertoire was represented as unique (V gene, J gene, CDR3 sequence) clonotypes, and each CDR3 was assigned the lowest Fisher's exact test p-value observed among its matching (V, J, CDR3) tuples. CDR3s absent from the training-derived statistics were assigned a p-value of 1.0. CDR3s were ranked in ascending p-value order, and top-ranked sequences were compared against the ground-truth driver CDR3s for that repertoire.

GIANA. Driver sequence scores were derived from the reference clusters produced by the GIANA training procedure. Within each fold, training sequences were clustered to form reference clusters, and test sequences were assigned to these clusters in GIANA's query mode. For each reference cluster, we computed the fraction of reference members originating from disease repertoires, excluding clusters with more than 100 reference TCRs. Each assigned test CDR3 received the disease fraction of its assigned cluster, while unassigned sequences or sequences assigned to excluded clusters received score zero. If the same CDR3 appeared in multiple cluster assignments, we retained the maximum disease-fraction score before ranking CDR3s in descending order.

DeepRC. Driver sequence scores were obtained from the trained sequence embedding and attention modules. Each CDR3 in a held-out repertoire was encoded using the same amino acid alphabet, positional features, and raw clone-count scaling used by the DeepRC dataloader, and then passed through the trained CNN sequence embedding network and attention network. We used the resulting attention logits to rank sequences, implemented as a softmax over all valid sequences in the repertoire. Duplicate CDR3s were aggregated by retaining the maximum softmax-normalized attention weight, and CDR3s were then ranked in descending order by this attention-derived score.

ABMIL. Sequence-level driver scores were taken from the trained gated-attention pooling module. For each held-out repertoire, all sequences were loaded without subsampling and converted to the model's amino acid, V-gene, and J-gene tensor inputs. The per-sequence encoder produced instance embeddings, which were passed through the gated-attention branches to obtain one pre-softmax attention score per sequence. These scores were softmax-normalized across all sequences in the repertoire. Duplicate CDR3s were aggregated by retaining the maximum attention weight. CDR3s were then ranked by descending attention weight.

VJ-kmer (Reg). Each unique (V gene, J gene, CDR3 sequence) tuple in a held-out repertoire was scored directly using the fitted logistic regression submodels. The k-mer submodel represented each CDR3 by its gapped 4-mer counts and applied the trained vectorizer, scaler, and logistic-regression `decision_function`, yielding a signed margin score. The V/J submodel represented each tuple by single-count V- and J-gene features and likewise applied the fitted vectorizer, scaler, and `decision_function`. The per-tuple driver score was the learned weighted combination of the margins from the two submodels, $\alpha s_{\mathrm{kmer}} + (1-\alpha)s_{\mathrm{VJ}}$. Duplicate CDR3s were aggregated by retaining the maximum score, and CDR3s were ranked in descending order.

VJ-kmer (XG). Each unique (V gene, J gene, CDR3 sequence) tuple in a held-out repertoire was scored directly using the fitted XGBoost submodels. The k-mer submodel represented each CDR3 by its gapped 4-mer counts and used the trained XGBoost booster to produce a disease probability under the `binary:logistic` objective. The V/J submodel represented each entry by single-count V- and J-gene features and likewise produced a disease probability. The per-entry driver score was the learned weighted combination of these probabilities, $\alpha p_{\mathrm{kmer}} + (1-\alpha)p_{\mathrm{VJ}}$. Duplicate CDR3s were aggregated by retaining the maximum score, and CDR3s were ranked in descending order.

bioRxiv preprint doi: https://doi.org/10.64898/2026.06.09.727013; this version posted June 10, 2026. The copyright holder for this preprint (which was not certified by peer review) is the author/funder, who has granted bioRxiv a license to display the preprint in perpetuity. It is made available under a CC-BY 4.0 International license.

B.8 Experiment setup for demographic confounding analysis

Demographic-matched cohort. For each of the four Mal-ID diseases analyzed in Figure 5 (left)—HIV, Lupus, Influenza, and Covid-19—we take a subset of the healthy repertoires matched on the cohort's dominant confounder. For HIV, we apply a *symmetric ancestry filter*: both the disease and healthy rows are restricted to specimens with self-reported African ancestry, the dominant ancestry group in the HIV cohort. For the three age-confounded diseases, we apply an *age-match-healthy* rule: the disease cohort is left unchanged and Healthy/Background is subsampled so that its 10-year-binned age histogram has the same shape as the disease cohort's. The matched healthy size N is the largest number consistent with the disease age histogram and the per-bin healthy availability; bins occupied by the disease but not the healthy pool are excluded. Each random-baseline cohort uses the *same disease side* as its matched run but replaces the healthy side with a uniformly random draw of the same N from the full healthy pool (ignoring demographics), isolating the effect of demographic structure from the cohort-size effect alone. We run five random-baseline replicates and report the mean AUROC. Within each (disease, cohort) configuration, classification follows the protocol of Section 4.1, reusing the preassigned 3-fold cross-validation splits.

Demographic-aware classification. For Figure 5 (right) we evaluate three representative methods—V/J gene (LogReg), ABMIL, and DeepTCR—on Lupus, HIV, and COVID-19. Influenza is excluded because age/sex/ancestry coverage in that cohort is too sparse to support paired comparisons. Within each panel, specimens missing any of {age, sex, ancestry} (or with empty ancestry) are dropped before any model is fit, so that the *Without demographics*, *With demographics added*, and *Demographics only* bars are evaluated on an identical specimen set. Demographic covariates are encoded as a numeric vector $[\text{age (raw)},\ \text{sex} \in \{0, 1\}\ \text{(binary)},\ \text{one-hot ancestry}]$, with the ancestry vocabulary fixed from the training fold and applied unchanged to the test fold. The *With demographics added* variant of each method appends this vector to the method's repertoire-derived features and trains a single L1-penalized logistic regression on the concatenated representation. The repertoire features are V/J gene relative-frequency counts (the V/J sub-model of the VJ-kmer ensemble) for V/J (LogReg), and the bag-level embeddings produced by a per-fold-trained ABMIL or DeepTCR model for the two deep-learning methods. Both feature blocks are standardized per fold before concatenation, and the corresponding *Without demographics* bar reuses the same pipeline but drops the demographic block. The *Demographics only* baseline (red dashed line) fits an L2-penalized logistic regression on the demographic feature vector alone, on the same specimen set. All three configurations use the predefined 3-fold CV splits, and we report pooled out-of-fold AUROC and AUPRC, consistent with Section 4.1.
